# Impact of modified radical neck dissections on the number of retrieved nodes, recurrence and survival

**DOI:** 10.1590/S1808-86942010000300017

**Published:** 2015-10-20

**Authors:** Hugo Fontan Kohler, Isabella Werneck da Cunha, Luiz Paulo Kowalski

**Affiliations:** 1 Physician; 2 Doctorate, medical pathologist, Pathology Department, A. C. Camargo Hospital; 3 Livre-Docente in onchology. Director of the Head & Neck and Otorhinolaringology Department, A. C. Camargo Hospital

**Keywords:** survival analysis, neck dissection, mouth neoplasms

## Abstract

Neck dissection is an integral part of head and neck tumors. Throughout its history, it has undergone changes looking for an improvement in functional outcome without loss of oncologic efficiency.

**Aim:** Demonstrate that the modified radical neck dissection have an oncologic results comparable to classical radical dissection.

**Materials and methods:** We included patients with squamous cell carcinoma of the lower floor of the mouth and oropharynx, who underwent radical classic or modified neck dissection. We excluded from this analysis those patients who had undergone previous treatment or extended neck dissection.

**Study design:** Retrospective study, involving an institution.

**Results:** We identified 481 patients who met the inclusion criteria, corresponding to 521 dissections. The average number of lymph nodes dissected was 44.92 (SD 16:45) lymph nodes to the RCT, 44.16 (SD 15.76) for the MRND + XI and 56.02 (SD 22.91) for the ECRM IJV + XI. The ANOVA indicated a statistically significant difference between groups (p<0.001). The type of neck dissection was not significant for regional recurrence or disease-specific survival.

**Conclusion:** The use of modified neck dissection has no significant impact on the pathological staging, disease-free survival or disease-specific survival.

## INTRODUCTION

Crille described radical neck dissection in 1906 as a procedure for en bloc removal of the sternocleidomastoid muscle, the internal jugular vein, the accessory nerve, and lymphatic and fatty tissues. The purpose of this procedure was to assure that most of the lymph nodes were removed, thereby maximizing its therapeutic effect and improving staging.^1^ Martin later defended this idea by arguing in favor of removing the abovementioned three non-lymphatic structures as a method for achieving full neck lymphadenectomy.^2^

Although full lymphadenectomy is achievable, classic radical neck dissection causes significant sequelae due to the removal of non-lymphatic structures. Thus, 40 years ago, efforts were made to preserve these structures – in particular the accessory nerve – when not affected by the tumor; these procedures became known as modified radical neck dissections.^3^ This trend continued, and methods were found to preserve the internal jugular vein and the sternocleidomastoid muscle, which resulted in the so called functional neck dissection.^4^,^5^

Notwithstanding the increasing popularity of these modified radical neck dissection procedures, a major issue is the impact of these modifications on surgical efficacy. We therefore investigated the number of recovered lymph nodes in each technique and the impact on regional recurrence and survival to test this effect.

## MATERIAL AND METHOD

This retrospective study investigated patients who had undergone neck dissection with the removal of lymphatic levels I to V, with or without removal of the accessory nerve, the internal jugular vein, or the sternocleidomastoid muscle. Patients with tumors of the lower mouth and oropharynx diagnosed histologically as squamous cell carcinoma were included. Patients who underwent extended neck dissections, preoperative radiotherapy or chemotherapy, or prior surgery were not included. For the statistical analysis patients were grouped according to the number of structures preserved into three groups: radical neck dissection (RND), neck dissection preserving the accessory nerve (MRND + XI), and neck dissection preserving the accessory nerve and the internal jugular vein (MRND IJV+XI). The AJCC 2002^6^ stating method was applied to all patients.

The Stata 10.1 (StataCorp, Texas, USA) software for MacOS X was used for the statistical analysis. Values are presented as the mean and standard deviation (SD). The analysis of variance (ANOVA) with Scheffé's test for multiple comparisons among groups was applied. The Kaplan-Meier and Cox methods were applied for assessing survival and regional recurrences; the hazard ratio (HR) value was indicated, and the confidence interval (CI) was 95%. Significant p values were p<0.05.

## RESULTS

There were 481 patients that fit the inclusion criteria. There were 424 (88.15%) males and 57 (11.85%) females. The mean age at the time of treatment was 55.97 years (SD 10.0 years). The most common primary site was the oral tongue (187 cases; 38.88%), followed by the floor of the mouth (126 cases; 29.19%), the retromolar trigone (69 cases; 14.35%), and the tonsils and lower gingival margin (44 cases; 9.15%). Table 1 shows the complete list of primary sites. Staging of the primary tumor revealed 19 T1 patients (3.95%), 202 T2 patients (42.0%), 148 T3 patients (30.77%), and 112 T4a patients (23.28%). There were 214 N0 patients (44.49%), 79 N1 patients (16.42%), 5 N2a patients (1.04%), 138 N2b patients (28.69%), 39 N2c patients (8.11%), and 6 N3 patients (1.25%). Bilateral radical neck dissection was carried out in 40 patients, resulting in a total 521 radical neck dissections. Contralateral dissection to the primary tumor was selective in 85 cases, and will not be analyzed in this study. Table 2 shows the distribution of neck dissections according to each group and laterality.

**Table 1:** Site of primary tumors in this series.

Tumor site	No. of patients (%)
Oral tongue	187 (38,88%)
Floor of the mouth	126 (29,19%)
Retromolar trigone	69 (14,35%)
Tonsil	44 (9,15%)
Lower alveolar ridge	44 (9,15%)
Base of the tongue	7 (1,45%)
Soft palate	2 (0,41%)
Jugal mucosa	1 (0,21%)
Posterior wall of the oropharynx	1 (0,21%)

**Table 2:** Distribution of neck dissections according to preserved structures and laterality to the primary tumor.

Type of neck dissection	Ipsilateral	Contralateral
Classic radical (group I)	336 (69,85%)	0 (0%)
MRND XI (group II)	91 (18,92%)	15 (37,5%)
MRND IJV + XI (group III)	54 (11,23%)	25 (62,5%)
Total	481 (100%)	40 (100%)

The mean number of dissected lymph nodes was 44.92 (SD – 16.45) in RND; 44.16 (SD – 15.76) in MRND +XI; and 56.02 (SD – 22.91) in MRND IJV+XI. ANOVA showed a statistically significant difference among groups (p<0.001), which was further investigated with the Scheffé test (Table 3); this test revealed that significantly more lymph nodes were recovered in patients undergoing modified neck dissections.

**Table 3:** Comparison of the number of recovered lymph nodes in each type of neck dissection.

	RND	MRND XI
MRND XI	-0.763 (p=0.932)	–
MRND IJV+XI	11.089 (p<0.001)	11.853 (p<0.001)

**Figure 1:**
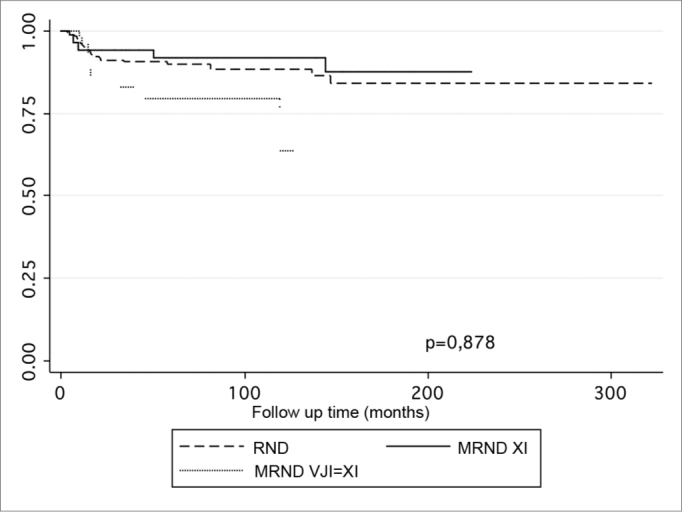
. Regional recurrence according to the type of radical neck dissection.

**Figure 2:**
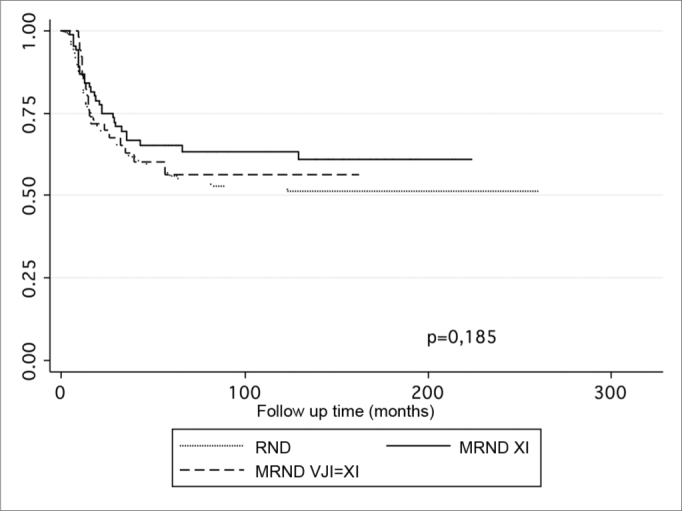
. Survival according to the type of radical neck dissection.

Follow-up ranged from 0.3 to 322.23 months (mean – 60.79 months, SD – 22.39 months). Local recurrence occurred in 122 cases; 74 of these were neck recurrences, and 46 were distance recurrences; 250 patients had recurrences in at least one site. Statistically significant factors for neck recurrences were age (p=0.037), staging of the neck (p=0.006), primary tumor thickness (p=0.002), and postoperative radiotherapy (p=0.046). In this model the type of neck dissection had no impact on regional recurrence (p=0.878, Table 4). Figure 1 shows the neck recurrence curves according to the type of neck dissection. Harrell's agreement test revealed a higher predictive ability of the model without including the type of neck dissection (Table 5). Statistically significant factors for disease-free survival were the T stage (p=0.001), the N stage (p=0.001), the presence of lymphatic vascular embolization (p=0.019), and tumor thickness (p=0.008). The type of neck dissection had no significant impact (p=0.185, Table 6). Survival curves on Figure 2 show a similar behavior in both groups. Harrell's agreement test showed that the model without the type of neck dissection was superior to the model with the type of neck dissection (Table 7).

**Table 4:** Statistically significant factors in a multivariate survival analysis of neck recurrence.

Variable	HR	95% CI	p
Age	1.0329	1.0020 – 1.0647	0,037
N stage	1.3917	1.0969 −1.7658	0,006
Thickness	1.0458	1.0165 −1.0760	0,002
Radiotherapy	0.4582	0.2086 −0.9875	0,046
Type of ND	0.9610	0.5795 – 1.5936	0,878

**Table 5:** Agreement analysis among regional recurrence models with and without applying the type of neck dissection.

Variables of the model	Harrell C	Somers D
Age, PN, thickness, radiotherapy	0,706	0,412
Age, PN, thickness, radiotherapy, type of ND	0,6904	0,3808

**Table 6:** Statistically significant factors in a multivariate analysis of disease-specific survival.

Variable	HR	95% CI	p
T stage	1,6221	1,2170 – 2,1622	0,001
N stage	1,2662	1,0984 −1,4597	0,001
Lymphatic embolization	1,9175	1,0984 – 3,3100	0,019
Tumor thickness	1,0326	1,0086 – 1,0572	0,008
Type of ND	0,7587	0,5044 – 1,1414	0,185

**Table 7:** Agreement analysis of disease-specific survival analysis with and without applying the type of neck dissection.

Variables of the model	Harrell C	Somers D
T stage, PN, lymphatic embolization, thickness	0,7456	0,4913
T stage, PN, lymphatic embolization, thickness, type of ND	0,7048	0,4817

## DISCUSSION

The extent of neck dissection has been an important topic for debates. Based on surgical and autopsy data, removal of lymphatic and fatty tissues without removing non-lymphatic structures was found to be oncologically sound. Neck lymph nodes are located within fibro-adipose tissue next to nerves and blood vessels; there are no lymph nodes in the muscle aponeurotic fascia.^4^ Bocca and Pignataro have confirmed these findings.^5^

Resection of the accessory nerve has been routinely avoided in selected cases since the 1950s because of the associated morbidity. It is preserved whenever there is a clear cleavage plan between the nerve and compromised lymph nodes.^7^

A criticism of modified radical neck dissections is the decrease in recovered lymph nodes relative to the number of preserved non-lymphatic structures. According to Busaba et al., classical radical neck dissection had a significantly higher number of recovered lymph nodes compared to the variations of this procedure.^8^ These authors suggested that this could have a negative impact on the prognosis of patients. However, Siddiquee et al. contested this finding by showing that classical and modified radical neck dissection had comparable oncological efficacy.^9^ This confirmed previously published papers that had also demonstrated a similar oncological efficacy of modified neck dissection, which has lower morbidity, especially in shoulder function.^10^

## CONCLUSION

Our series showed that the modified radical neck dissection has a similar oncological efficacy to the classical radical neck dissection, and that contrary to previous arguments in the literature, the number of recovered lymph nodes is higher than that in classical neck dissection. There is thus no loss in staging on pathology and the prognosis of patients, or in their survival and disease-free interval.
